# Shoulder diagnoses in secondary care, a one year cohort

**DOI:** 10.1186/1471-2474-15-89

**Published:** 2014-03-18

**Authors:** Niels G Juel, Bård Natvig

**Affiliations:** 1 Department of Physical Medicine and Rehabilitation, Oslo University Hospital, Ullevål, Postboks 4956 Nydalen, 0424 Oslo, Norway; 2 Department of General Practice, Institute of Health and Society, University of Oslo, Oslo, Norway

**Keywords:** Shoulder, Diagnosis, Prevalence, Hospital outpatient clinics, Age factors

## Abstract

**Background:**

Shoulder pain is common in the general population. Reports on specific diagnoses in general populations are scarce and only from primary care. The diagnostic distribution of shoulder disorders in secondary care is not reported. Most of the clinical research in the shoulder field is done in hospital settings. The aim of this study was to identify the diagnoses in a 1-year cohort in a hospital-based outpatient clinic using standardized diagnostic criteria and to compare the results with previous studies.

**Methods:**

A diagnostic routine was conducted among patients referred to our physical medicine outpatient clinic at Oslo University Hospital. Diagnostic criteria were derived from the literature and supplemented with research criteria.

**Results:**

Of 766 patients diagnosed, 55% were women and the mean age was 49 years (range 19–93, SD ± 14). The most common diagnoses were subacromial pain (36%), myalgia (17%) and adhesive capsulitis (11%). Subacromial pain and adhesive capsulitis were most frequent in persons aged 40–60 years. Shoulder myalgia was most frequent in age groups under 40. Labral tears and instability problems (8%) were most frequent in young patients and not present after age 50. Full-thickness rotator cuff tears (8%) and glenohumeral osteoarthritis (4%) were more prevalent after the age of 60. Few differences were observed between sexes. We identified three studies reporting shoulder diagnoses in primary care.

**Conclusion:**

Subacromial pain syndrome, myalgia and adhesive capsulitis were the most prevalent diagnoses in our study. However, large differences in prevalence between different studies were found, most likely arising from different use of diagnostic criteria and a difference in populations between primary and secondary care. Of the diagnoses in our cohort, 20% were not reported by the studies from primary care (glenohumeral osteoarthritis, full thickness rotator cuff tears, labral tears and instabilities).

## Background

Shoulder pain is common in the general population with a reported 1-month prevalence of 18–31% and a 1-year prevalence of 5–47% [1-3]. These reports were questionnaire-based from unselected community populations. The focus was on pain in the shoulder region reported with varying case definitions. Reports on specific diagnoses in general populations are scarce. We found three studies in this field using an unsystematic literature search in PubMed and browsing bibliographies of relevant articles. Walker-Bone *et al.*[4] invited all persons with shoulder pain in a survey to a clinical appointment, where the subjects were given diagnoses using a standardised clinical examination. The two largest diagnostic groups by far were adhesive capsulitis and rotator cuff tendonitis at 55% and 30% respectively, giving a 1-year prevalence of 9% and 5% of these diagnoses in the general population. Two prospective diagnostic studies from general practice [5,6] were also using standardised tests to obtain diagnoses. Östör *et al.* found rotator cuff related disease to be the most common diagnosis, and it represented up to 86% of all cases whereas adhesive capsulitis represented 15%. Van der Windt *et al.*[6] found that 44% of the diagnoses were rotator cuff related and found adhesive capsulitis in 21% of the population. These numbers show large differences in the reported proportions of the various diagnoses in different studies. These differences might be caused by the different populations examined but also by the various sets of diagnostic criteria used. Most of the shoulder diagnoses are based on clinical criteria. Diagnoses such as osteoarthritis, rotator cuff and labral tears also rely mainly on reported pain and positive clinical tests to be confirmed as possible reasons for the patient’s pain, although they are in some cases image supported [7]. This is particularly the case in persons aged over 60 years because of the natural and mostly pain-free changes in the tissue that come with age [8-10].

To our knowledge, the diagnostic distribution of shoulder disorders in secondary care is not reported. Most of the research in the shoulder field is done in hospital settings. If the majority of diagnoses are the same in general practice as in a specialist outpatient clinic, the generalisability of results from research done in specialist practices will tend to increase. The same might be true for diagnostic measures. Therefore, we considered it would be interesting to compare the diagnostic distribution in primary care with diagnoses given in a specialist practice.

The aim of this prospective study was to identify the diagnoses in a 1-year cohort in a hospital-based outpatient clinic using standardized diagnostic criteria and to compare the results with previous studies.

## Methods

### Patient selection

The data in this article were collected routinely from an outpatient clinic at the Department of Physical Medicine and Rehabilitation in Oslo University Hospital, Ullevål, Norway. Referrals to the clinic come mainly from primary care physicians.

All referrals regarding shoulder pain were marked with the World Health Organization International Classification of Diseases (ICD)-10 diagnosis m25.51 “Shoulder joint pain” in the hospital’s administrative system (DoculivePasDoc 3.0, Siemens, Munich, Germany). All patients were examined by a medical doctor who took a history and performed a systematic clinical examination. When considered helpful, diagnostic ultrasound, X-rays, MRI, blood samples or neurophysiological investigations were conducted in addition before the final ICD-10 diagnosis was decided and recorded. The registration period started on 1 April 2008 and ended on 31 March 2009, giving a 1-year cohort.

The data were extracted from DoculivePasDoc through a report of all referrals marked m25.51. This report contained the patient’s ID, date of visits, the final ICD-10 diagnosis given, as well as gender and duration of pain. The data were de-identified after review of the records. As the study was part of regular clinical activity and patient privacy was routinely taken into account, informed consent and permission from the Regional Ethics committee was not considered necessary.

### Clinical examination and diagnostic criteria

Five medical doctors worked in the clinic during the registration period: four were specialists in physical medicine and rehabilitation and one was undergoing speciality training. Diagnostic criteria for the various diagnoses were established in accordance with the literature [8,10-17] as listed in Table 1. The diagnostic criteria was based on an unsystematic literature search of PubMed and references cited in the captured articles.

**Table 1:** Symptoms and diagnostic criteria for the most common shoulder diagnoses according to the ICD-10 code

**ICD-10 diagnosis with description**	**Symptoms**	**Diagnostic criteria**	**Radiologic investigations that might increase diagnostic accuracy**	**Number (% of all) in this study**	**References**
**M75.4**	Pain in the shoulder and proximal lateral upper arm exacerbated by activity	Typical pain **and** positive impingement test **and** pain with isometric abduction or external rotation	None	275 (36)	[11-13]
Subacromial pain syndrome					
**M79.11**	Diffuse pain outside the glenohumeral (GH) joint localised over muscles	Negative specific tests, pain when palpating muscles	None	132 (17)	[11]
Myalgia in shoulder muscles					
**M75.0**	Pain in the shoulder exacerbated by activity. Feeling of stiffness.	Reduced passive range of GH motion >30° in two planes	None	86 (11)	[14,15]
Adhesive capsulitis					
**M75.1**	Pain in the shoulder. Occasional feeling of weakness	Positive impingement test **and** weakness with isometric abduction or external rotation	MRI and US	58 (8)	[8,16]
Full thickness rotator cuff tear					
**M19.8**	Pain on top of shoulder, over the AC joint	Pain with joint palpation. Osteoarthritis on X-ray, US or MRI	X-ray	31 (4)	[11,18]
Acromioclavicular (AC) joint osteoarthritis					
**M19.0**	Pain in the shoulder. Occasional feeling of stiffness	Osteoarthritis on X-ray or MRI	X-ray	29 (4)	[10,11]
Glenohumeral (GH) joint osteoarthritis					
**M24.3**	Pain in the shoulder and/or feeling of instability	Positive apprehension/relocation test. Labral tear on MRA	MRA	24 (3)	[11]
Anterior labral tear or instability					
**M24.3**	Pain in the shoulder. Occasional feeling of instability	Positive O’Brian test and SLAP lesion on MRA	MRA	16 (3)	[11]
SLAP lesion					
**M25.2**	Pain in and around the shoulder. Occasional feeling of instability	Positive sulcus sign and passive range of GH external rotation >90°	None	9 (2)	[11]
Multidirectional instability					

Main symptoms, diagnostic criteria and radiological investigations that might have increased the accuracy of the diagnostic criteria are listed with and references for the used criteria.

Key: MRI, magnetic resonance imaging; MRA, MRI arthrography; SLAP, superior labrum anterior to posterior; US, ultrasound.

The numbers (%) of patients diagnosed with different diagnoses are shown.

The criteria were implemented prior to the data registration period. Training sessions in performing the clinical tests needed for diagnostic purposes were conducted to increase inter rater reliability which is reported to be varying from slight to substantial for the tests used, see Table 2. Some of the diagnoses were supported by supplementary imaging such as x-ray (osteoarthritis), MRI (where clinical examination were inconclusive), MRI arthrography (labral tears) or ultrasound (full thickness tears). However, most diagnoses were essentially based on the patient’s medical history and a combination of clinical tests. The examiners followed the diagnostic scheme shown in Table 1 when diagnosing the patients. The diagnosis myalgia was chosen when specific diagnoses from the joints, rotator cuff or labrum were excluded, the patient felt pain over the muscles and the examiner triggered recognizable pain in the area by palpation. Reinforcement of the diagnostic routines was done regularly during the data collection period. Discussions concerning unclear cases were held in the clinic as well when needed. The tests used in the diagnostic procedures in Table 1 and their reliabilities are listed in Table 2.

**Table 2:** The reported reliability of the clinical tests used in the diagnostic procedure

**Test**	**Inter rater reliability. ICC or Kohens kappa (K). (95% CI) or mean.**	**Reference**
*Passive glenohumeral range of motion*		
- External rotation	0.88-0.90, 0.87-0.93	[19,20]
- Abduction	0.84-0.87, 0.85-0.98	[19,21]
*Isometric muscle tests*		
- Abduction (empty can)	0.30-0.94 (K)	[22]
- External rotation	0.37-0.90 (K)	[23]
- Belly press	0.61 (K)	[24]
*Special tests*		
Hawkins impingement test	0.18-0.91 (K), 0.38 (K)	[22,24]
AC joint palpation	na, high specificity (0.73)	[17,24]
Apprehension test	0.31-0.47	[22]
Relocation test	0.31-0.71	[22]
O´Brien test	0.24-0.38 (K)	[24]
Sulcus sign	0.60	[22]

### Statistical analysis

The data were analysed using the statistical package SPSS v. 15.0 (SPSS Inc., Chicago, IL, USA). Descriptive statistics (mean, range and standard deviation, SD) were calculated. The relative proportion of one diagnosis (% of all diagnoses) in an age group was calculated by dividing the number of patients with this diagnosis in that age group (nd-ag) with all patients in the same age group (n-ag) times 100; nd-ag/n-ag*100.

## Results

During the 1-year registration period, 766 patients were referred to the clinic with a shoulder problem. Fifty-five per cent of the patients were women (419/766) and overall mean age in both sexes were 49 years (range 15–93; SD ± 14). The mean duration (± SD) of shoulder pain was 29 ± 42 weeks for men and 26 ± 38 weeks in women. 471 (61%) of the patients supplied a compact disc with a magnetic resonance imaging (MRI) scan or had a description of the scan attached to their referral letter.

**Figure 1:**
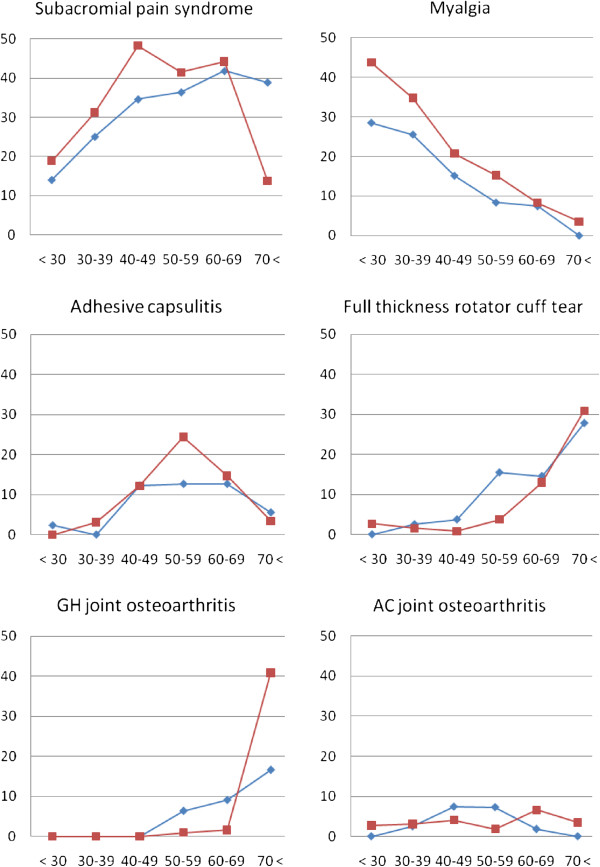
Percentages of the six most common shoulder diagnoses among six age groups in women (red squares, dotted line) and men (blue diamonds).

The most common diagnoses were subacromial pain syndrome (36%), followed by myalgia (17%), adhesive capsulitis (11%), full thickness rotator cuff tears (8%), acromioclavicular (AC) joint osteoarthritis (4%) and glenohumeral (GH) joint osteoarthritis (4%). The relative proportions of these diagnoses are shown in Figure 1.

Subacromial pain was most frequent in patients aged between 40 and 60 years. For men, this diagnosis increased with age, but in women it was reduced after age 70. For myalgia, most affected individuals were also aged 40–60. The relative proportion of this diagnosis was highest in the lowest age group, 44% in women and 29% in men, decreasing almost linearly with age to less than 5% in both women and men aged over 70. Adhesive capsulitis was most frequent between 40 and 70 years of age for both men and women, as was the case for the proportion of this diagnosis. For full-thickness rotator cuff tears and glenohumeral osteoarthritis, almost all affected individuals were aged over 50 years. The proportion of both full-thickness rotator cuff tears and glenohumeral osteoarthritis increased dramatically from less than 5% before 50 years, up to 30% of all patients over 70 except for men with osteoarthritis (17%). Acromioclavicular osteoarthritis was most frequent for patients aged 40–70.

**Figure 2:**
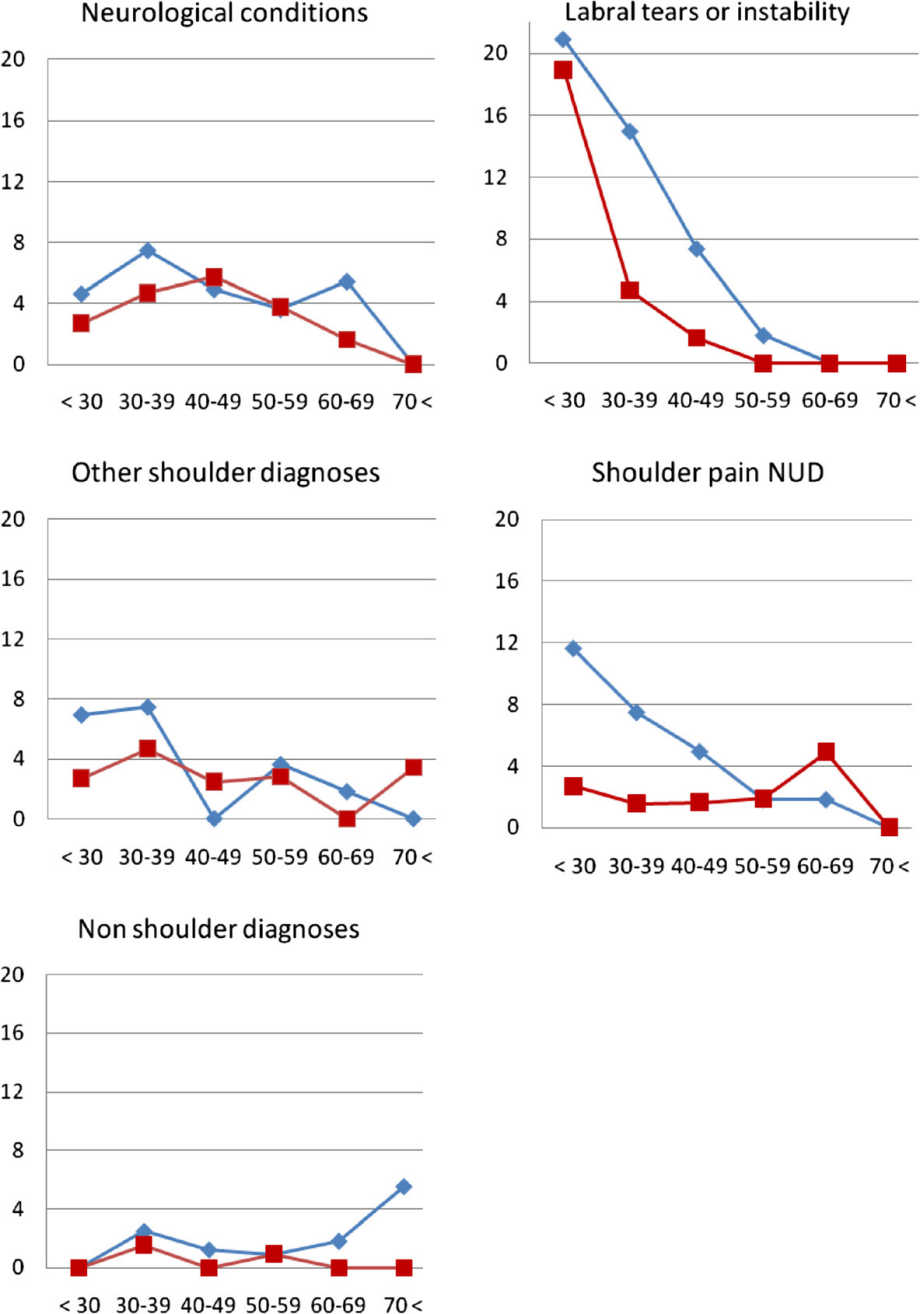
**Percentages of less frequent shoulder diagnoses and non-shoulder diagnoses among six age groups in women (red squares, dotted line) and men (blue diamonds).** The Y-axis covers 0–20% of all cases by age group.

The remaining diagnoses were grouped into labral tears (6%), neurological conditions (4%), other shoulder diagnoses (5%) and non-shoulder diagnoses (1%). Of the study cohort, 26 patients (3%) did not get a specific diagnosis and were labelled “shoulder pain NUD”. Of the 49 patients with labral tears, 19 had anterior tears and five had posterior tears. Sixteen had superior labrum anterior to posterior (SLAP) lesions and nine were diagnosed with multidirectional instability. There were 31 patients with neurological conditions. Of these, 17 had cervicobrachialgia without radiculopathy, five had radiculopathy and nine suffered from mononeuritis in the shoulder girdle. Among other shoulder diagnoses, we found 10 sequelae after fracture, seven with AC-joint rupture and seven with biceps tendinopathy only. There were one to three patients with the following diagnoses: sternoclavicular joint pathology, avascular necrosis of the caput humeri, snapping scapulae, postoperative pain, neuropathic pain, septic bursitis and generalised hypermobility syndrome. The eight non-shoulder diagnoses included pulmonary tumour, polymyalgia rheumatica, thoracic back pain, psoriatic cervical osteochondritis, myopathy and monoarthritis in the elbow. The relative proportions of the less frequent diagnoses labral tears, neurological conditions, other shoulder diagnoses and non-shoulder diagnoses are shown in Figure 2 on a different scale than the common diagnoses shown in Figure 1.

## Discussion

### Main findings

Subacromial pain syndrome was the most common shoulder diagnosis (36%) and it increased with age for both men and women. Then myalgia in the shoulder girdle (17%) and adhesive capsulitis (11%) followed. Myalgia was the most frequent diagnosis in persons under 40 years for both genders, decreasing linearly with age. Adhesive capsulitis was found almost entirely in patients aged 40–70 with an even sex distribution except for a peak in women between 50 and 59 years. The degenerative diseases full thickness rotator cuff tear (8%) and glenohumeral osteoarthritis (4%) represented fewer patients, but accounted together for almost 60% of the patients over 70 years of age. In men between 50 and 69 years, glenohumeral osteoarthritis was six times more frequent than in women, among whom the diagnosis was present almost entirely after the age of 70 years.

### Methodological aspects

The results in this study were collected from routine diagnostic practice in an outpatient clinic. The practice was based on a literature-based written diagnostic consensus reinforced by regular supervision, meetings on a regular basis and discussions regarding difficult cases. The diagnostic criteria listed in Table 1 are mainly based on symptoms and clinical investigations and only supported by imaging in three of our nine diagnostic categories. However, most of the patients either brought (471 (61%)) or had imaging, mostly with MRI, but also with X-rays and diagnostic ultrasound. This supplementary use of imaging might be a problem in this study, as imaging give many incidental findings not necessarily contributing to the symptoms. However, this study lacked the rigid framework of a scientific study with a systematic pre-approved registration. There was no quality assessment of potential differences between the involved consultants. Intra- and inter rater reliability measures on the performance of the clinical tests were not obtained, although training before the registration period was done. The lack of inter rater reliability testing is an important limitation of this study and might lead to variability in prevalences of the selected diagnoses.

For 17 patients, the pre-set diagnosis was not changed to a more specific diagnosis after the consultation due to lacking registration from the consultants. To include these patients the senior consultant (NGJ) corrected these diagnoses by going through these medical records after the registration period, and retrospectively set a diagnosis according to the methods used in this study.

The most common diagnoses of shoulder ailments are based on sets of criteria in the absence of tissue specific characteristics. There is no conclusive gold standard and the pain generator is not known for conditions such as subacromial pain syndrome and myalgia. This is even true in the degenerative diseases where studies have shown both full thickness rotator cuff tears [8] and osteoarthritis [9] in non-painful shoulders. Therefore, the diagnostic criteria sets used in the literature might differ and cause frequency differences across studies. Our choice of criteria sets was mostly based on the Southampton examination schedule [11,25], and additional research-based criteria were added when considered adequate (Table 1). The myalgia diagnosis is disputed and argued to be a symptom and not a specific diagnosis. In this study myalgia is a symptom diagnosis supplemented with positive findings by muscle palpation.

We report only one main diagnosis per person although some patients had more than one diagnosis. This have probably affected the results, particularly in the myalgia and AC joint osteoarthritis groups. AC joint osteoarthritis is reported as increasingly present with age in persons without shoulder pain [9] and was most likely underestimated in older age groups in our study. Myalgia is almost always present in joint diseases as an additional extra-articular source of pain but was only accounted for when present as the main diagnosis in our cohort. Probably myalgia and AC joint osteoarthritis would have been among the most prevalent secondary and tertiary diagnoses, but due to the lack of registration of more than one main diagnosis this study can not present results on the distribution of secondary and tertiary diagnosis.

### Discussion of our results compared with other reports

A search of the literature did not reveal any studies reporting the prevalence of shoulder diagnoses in secondary care. Three studies reporting shoulder diagnoses from population studies or primary care were identified. Walker-Bone *et al.*[4] (447 shoulders) recruited patients from a questionnaire study of 9696 persons in the general population and 365 shoulder diagnoses were made. Östör *et al.*[5] investigated a 1-year cohort from two general practices (131 shoulders). These two studies were from England, whereas van der Windt *et al.*[6] described a Dutch 1-year cohort (392 shoulders) recruited from 11 general practitioners.

The percentages of the six most common diagnoses in these three population studies are shown in Table 3 together with percentages from our study.

**Table 3:** Percentages of the most common diagnoses in four studies

	**This study**	**Walker-Bone **[4]	**Östör **[5]	**Van der Windt **[6]
Subacromial pain syndrome	36	30^a^	86^b^	44^c^
Adhesive capsulitis	11	55	15	21
Myalgia	17	–	6	–
Full thickness rotator cuff tear	8	–	–	–
Acromioclavicular osteoarthritis	4	6	31	5
Glenohumeral osteoarthritis	4	–	–	–

^a^Includes rotator cuff tendonitis and subacromial bursitis.

^b^Includes impingement and rotator cuff tendinopathy.

^c^Includes tendonitis and chronic bursitis.

Walker-Bone *et al.* reported from a population-based study in which the participants complained of shoulder pain in the previous week when asked, but did not seek help themselves. This population probably had fewer complaints regarding both duration and pain intensity compared to the other two studies that are based on patients seeking help for their shoulder symptoms. It is surprising that Walker-Bone *et al*. found adhesive capsulitis in 55% of the cases because this is a disease regarded to cause a lot of pain and restriction in function. In contrast the other two studies found 15% and 21% of this diagnosis.

The subacromial pain syndrome diagnosis was separated into subdiagnoses in the other studies as impingement and rotator cuff tendinopathy [5], rotator cuff tendinitis and subacromial bursitis [4] or as tendonitis and chronic bursitis [6], giving the opportunity of multiple diagnoses in the same person. This might have affected the reported numbers of patients with subacromial pain syndrome. Separation of specific diagnoses in the subacromial area is disputed, both on the grounds of clinical tests and of radiological investigations [26,27]. Therefore, in our study, we chose to collect all patients with subacromial pain and no other specific tissue diagnosis as listed above into one group called subacromial pain syndrome. Östör *et al.* reported subacromial pain in 86% of their subjects, double the rate of the other studies. Their criteria included discomfort with isometric testing of any of the rotator cuff muscles. Isometric testing gives a co contraction of all cuff muscles and this will increase the compressive forces in the GH joint, engage the trapezius and scapular muscles and may trigger pain from capsulitis in the GH joint or from painful muscles [14]. Isometric tests with the arm elevated produce shear forces in the AC joint [28]. Pain during isometric testing might have led to overdiagnosing subacromial pain.

Surprisingly, none of the other studies reported full thickness rotator cuff tears or glenohumeral osteoarthritis. In our study, 14% of all diagnoses in the 60–69 age group were tears, rising to 30% in persons over 70 years of age. Glenohumeral osteoarthritis was the most frequent diagnosis in women aged over 70 years (41%) and accounted for 17% of the diagnoses in men of this age group. This large difference in results between the four studies might occur because these degenerative diagnoses are rare in primary care. On the other hand, one would expect scattered cases in a 1-year cohort with ages up to 87 years. In our cohort, all diagnoses of glenohumeral osteoarthritis and full thickness rotator cuff tears were supported by MRI scans, which have very high sensitivity for these diagnoses [29]. This might account for a higher proportion of these diagnoses in our group. On the other hand full thickness cuff tears is frequently found also in non-painful shoulders in the elderly [30] and may have led to overdiagnosing these conditions in our population. However, there were only 28 patients with osteoarthritis and full thickness tears in our cohort of 766. The other cohorts in the literature were smaller than ours and a random distribution of diagnoses may also explain some of the variation in the results.

In the present study, AC joint osteoarthritis had to fulfil the clinical criteria in Table 1 and to be the main diagnosis to be registered. In clinical practice, symptomatic AC-joint osteoarthritis often coexists with, or is a part of, a subacromial pain syndrome. Therefore, the number of AC-joint osteoarthritis diagnoses in our study might have been underestimated because subacromial pain was chosen most often as the main diagnosis. Östör *et al.* found a high percentage of AC joint osteoarthritis compared with the other studies. This might be because their diagnostic criteria included either tenderness in the AC joint region or pain on adduction of the arm only. Adduction is often painful in patients with subacromial pain syndrome and always in cases of adhesive capsulitis and this may have led to an overestimation of AC joint osteoarthritis in their study.

Walker-Bone *et al*. did not find any age or gender-related difference in diagnoses, and the other reports did not detail such differences. In our study, the most striking difference was the approximately 10% higher proportion of myalgia in women aged up to 60 years, a higher proportion of subacromial pain syndrome in women under 50 years of age and a higher proportion of full thickness tears after the age of 60 for both sexes.

In terms of the duration of pain, there were only one comparable study from primary care [6]. Östör *et al.* reported the median duration of pain to be 10 weeks. The participants in our cohort had suffered from shoulder pain for 26 weeks on average at the consultation. This might imply a more chronic group in secondary care, which is also to be expected. The rather large difference in the frequencies of the diagnose myalgia might be explained by the different diagnostic criteria used in the studies but it could also partly reflect the longer duration of pain in secondary care.

The generalisability of this study is limited because of differences in the spectre of diagnoses used compared to other studies, and also the methodological limitations with the lack of inter rater reliability testing, the missing secondary and tertiary diagnoses and the pragmatic use of clinical tests and imaging as diagnostic tools.

However, our data is the first description of the shoulder diagnoses in a secondary care setting, and we found that the most frequent diagnoses were subacromial pain, myalgia and adhesive capsulitis. Our results from secondary care are partly in line with studies from primary care settings. In all these studies subacromial pain and adhesive capsulitis represented 50% or more of the cases for both men and women. Consequently, these two diagnoses should always be considered in patients presenting with shoulder pain in all levels of health care.

## Conclusions

This is the first report on the prevalence and age and gender related prevalence of specific shoulder diagnoses in secondary care. The most frequent diagnoses set in our clinic were subacromial pain syndrome, myalgia in the shoulder girdle and adhesive capsulitis. Subacromial pain syndrome and adhesive capsulitis were most frequent in the middle age and the degenerative diseases were almost only present after the age of 60 and represented 60% of all diagnoses in persons over 70. In people under 40 myalgia and instabilities were most frequent.

## Competing interests

The authors declare that they have no competing interests.

## Authors’ contributions

NGJ planned and designed the study, took part in all phases of the study and are responsible for the work as a whole. BN contributed in analysis and interpretation of data, drafting and revising the manuscript and approved the final submitted version. Both authors read and approved the final manuscript.

## Authors’ information

NGJ is head of the outpatient clinic for musculoskeletal diseases and responsible for the quality of diagnostic procedures in his department and also for specialist education in the field in Norway. BN is professor in general practice and do research on the epidemiology of musculoskeletal pain.

## Pre-publication history

The pre-publication history for this paper can be accessed here:

http://www.biomedcentral.com/1471-2474/15/89/prepub
